# Relationship between Musical Characteristics and Temporal Breathing Pattern in Piano Performance

**DOI:** 10.3389/fnhum.2016.00381

**Published:** 2016-07-28

**Authors:** Yutaka Sakaguchi, Eriko Aiba

**Affiliations:** ^1^Graduate School of Informatics and Engineering, University of Electro-CommunicationsChofu, Japan; ^2^Center for Art and Performance Science, University of Electro-CommunicationsChofu, Japan

**Keywords:** respiratory control, breathing rate, music performance, musical phrasing, inter-trial consistency, individual differences

## Abstract

Although there is growing evidence that breathing is modulated by various motor and cognitive activities, the nature of breathing in musical performance has been little explored. The present study examined the temporal breath pattern in piano performance, aiming to elucidate how breath timing is related to musical organization/events and performance. In the experiments, the respiration of 15 professional and amateur pianists, playing 10 music excerpts in total (from four-octave C major scale, Hanon's exercise, J. S. Bach's Invention, Mozart's Sonatas, and Debussy's *Clair de lune*), was monitored by capnography. The relationship between breathing and musical characteristics was analyzed. Five major results were obtained. (1) Mean breath interval was shortened for excerpts in faster tempi. (2) Fluctuation of breath intervals was reduced for the pieces for finger exercise and those in faster tempi. Pianists showing large within-trial fluctuation also exhibited large inter-excerpt difference. (3) Inter-trial consistency of the breath patterns depended on the excerpts. Consistency was generally reduced for the excerpts that could be performed mechanically (i.e., pieces for finger exercise), but interestingly, one third of the participant showed consistent patterns for the simple scale, correlated with the ascending/descending sequences. (4) Pianists tended to exhale just after the music onsets, inhale at the rests, and inhibit inhale during the slur parts. There was correlation between breathing pattern and two-voice polyphonic structure for several participants. (5) Respiratory patterns were notably different among the pianists. Every pianist showed his or her own characteristic features commonly for various musical works. These findings suggest that breathing in piano performance depends not only on musical parameters and organization written in the score but also some pianist-dependent factors which might be ingrained to individual pianists.

## Introduction

Breathing is tightly coupled with various human activities, despite the fact that the major purpose of breathing is gas exchange (i.e., oxygen uptake and carbon dioxide ejection). In the first place, breathing is realized by the contraction and relaxation of the diaphragm and the movement of the thorax (e.g., Ratnovsky et al., 2008), meaning that the breathing cannot be independent from our motor control of body movement. On the other hand, most methods of meditative practice (including yoga) tell us to steady our breathing for meditation, implying that breathing is also related to our mental activities.

Such coupling between breathing and various human activities has been investigated from a scientific viewpoint (Bartlett and Leiter, 2012 for review). The entrainment of breathing by the rhythm of body exercise can be observed in bicycle exercise and walking on a treadmill (Bechbache and Duffin, 1977; Loring et al., 1990). Synchronization between locomotive movement and breathing is also observed in running, though the synchronization of gait and respiration is not observed for inexperienced runners (Bramble and Carrier, 1983). Coordination between breathing and gait is also observed in walking: the degree of coordination is enhanced with increasing walking speed, but only slightly with increasing work load (Rassler and Kohl, 1996). Furthermore, breathing can be entrained by rhythmical manual tracking movements (Ebert et al., 2000). The coordination between breathing and tracking movements occurs at different rate ratios depending on the movement rate. Similar entrainment is also observed with finger tapping synchronized with visual signals (Wilke et al., 1975).

These facts clearly show that our respiration rhythm is often coupled with the rhythm of body movement; likewise, breathing is modulated by other human activities. Boiten (1993) examined the respiratory patterns of human participants performing four mental and physical tasks (mental arithmetic task, sustained attention task, relaxation task, and four levels of graded exercise), and found that different tasks exhibited distinct breathing patterns characterized by the cycle duration and duty ratio. In addition, breathing can be entrained by listening to music (e.g., Haas et al., 1986; Bernardi et al., 2006). Recently, moreover, the relationship between emotion and breathing has grabbed the attentions of various researchers (Boiten et al., 1994; Boiten, 1998; Holstege et al., 2014).

Therefore, previous studies show that breathing is coupled with our physical, cognitive, and emotional activities. This suggests that multiple neural mechanisms are involved in breath control. Basically, respiratory movements occur automatically and continuously, driven by neural circuits (i.e., central pattern generators) within the brainstem and spinal cord (Smith et al., 1991, 2013; Feldman and Kam, 2015). This network does not only receive modulatory inputs from supra-brainstem structures (including motor and sensory cortices), but also sends signals to the cortex (Pattinson et al., 2009). Recent research indicates that brain activity may be linked to respiration (Ito et al., 2014) therefore, the coupling between breathing and other human activities might plausibly stem from the interaction between the respiratory center in the brain stem and various functional modules in the cerebral cortex.

Breathing has also been recognized as a significant factor affecting human performance in many practical fields. In the field of music, for example, breathing is commonly considered to be related to performance. According to many pianists, teachers sometimes give instructions on breathing (e.g., Gat, 1968; though the musicians' “breathing” might not mean the physiological respiration). This fact suggests that breathing in piano playing is an important factor to performance. The primary purpose of the present study is to examine this empirical hypothesis by a systematic experiment, and to assemble a picture of breathing in music playing.

Many researchers have investigated the nature of breathing when playing musical instruments. In studies on those instruments whose performance is directly linked to respiratory function, Bouhuys (1964, 1969) examined the breathing patterns of 42 professional wind-instrument players. He found that wind-instrument players generally displayed common typical pattern of rapid inspirations followed by prolonged and discontinuous expirations though the detailed pattern differed depending on the instruments. In contrast with wind-instrument players who have to keep expiring to make sounds, pianists are free from such limitations, and this fact leads us to a question what factors affect the breath timing in piano playing.

Several studies have examined this question. Ebert et al. (2002) asked six pianists to perform a finger exercise with different types of meters (3/4, 4/4, …, 7/4) and examined the temporal relationship between the onsets of finger strokes and inspirations. Playing tempo (finger-beat-rate), chosen by each pianist, was almost constant between different meter conditions. Their major finding was that the ratios of meter and breathing rates clustered around different integer values depending on the type of meter, suggesting that the mental grouping of notes by musical meters interacted with breathing rhythm. They also found that mean breathing frequency in piano performance was higher than in resting (breathing rate increased as soon as the pianists started playing), and that breathing period was more variable in piano playing than at rest.

Nassrallah et al. (2013) also examined the relationships of respiration and finger movements when eight pianists played the C major scale and arpeggio at different tempi. The breathing varied between participants and, in some cases, the timing of specific movement points (e.g., meter and thumb passage) coincided with the respiration. Generally, however, no coordination occurred between breathing and finger movement, not in line with the results of Ebert et al. (2002).

King (2006) ran a pilot experiment where pianists' breathing was monitored in relation to music-structural gestures and pianists' physical movements. She asked three pianists to perform three musical pieces (Bach, Beethoven, and Poulenc). The pianists played each piece twice. Mean breath intervals differed between participants and between musical pieces (except for one participant) although they were consistent between two performances of the same piece. Breath rates in piano playing were comparatively higher than the base rate, but musical tempo did not necessarily influence breathing rate. Moreover, type of breath (inspiration vs. expiration) at the start and end of performances and at the beginning and ending of phrases varied depending of pianists and musical pieces. Pianists' breathing was fundamentally unique in each performance, but some common tendencies were found in places: all of the performances were initiated by a “preparatory breath” that occurred before the downbeat or first note of a piece and 72% of these breaths were inhalations. She also analyzed the relation between breathing and physical movements (e.g., body sway, hand lifts, and head tilts). On the basis of these results, she argues that breathing is somehow linked to musical tempo, music-structural gestures, and pianists' physical movements though the connection is primarily unconscious and extremely flexible.

As a study on the emotional effect of piano performance, Nakahara et al. (2010) compared several autonomic and cardio-respiratory parameters between expressive piano performance and not-expressive one. Breath frequency and tidal volume were modulated by pianists' emotional state. In a separate study, the same group (Nakahara et al., 2011) showed that the emotional modulation in the psychophysiological responses was greater for the music players (i.e., pianists) compared with the music listeners (listening to the same piece of music).

Therefore, the nature of the breathing of piano players has been already examined from a scientific viewpoint. However, most of these studies have focused on the effect of some specific musical parameters (e.g., tempo and meter) and the players' state (e.g., emotion); few studies have examined the relationship between musical events/organization and temporal breathing patterns. Exceptionally, King (2006) examined this issue, but her pilot experiment was done only with a few participants and a few musical pieces.

In the present study, we measured the temporal respiratory patterns of 15 professional and amateur pianists playing 10 music excerpts, and analyzed their relationship with the musical characteristics. We mainly asked (1) how the breath interval and its variance depended on the musical works, (2) whether the breathing pattern was uniform over the musical work or related to the musical events (e.g., music onset, rest and slur), (3) whether the breath patterns were consistent between different trials, and (4) whether different pianists showed similar breath patterns or their own specific patterns.

Preliminary results of this study have been presented elsewhere (Sakaguchi and Aiba, 2015).

## Methods

### Participant

Fifteen active Japanese pianists (2 were male and 13 were female), including one of the authors, participated in the experiments. Their ages ranged from 21 to 50 years (mean: 31.7 years, standard deviation: 9.9 years). Seven of the participants were professional performers and piano teachers (all have received professional education in college), six were graduate and undergraduate students majoring in piano performance in the School of Music (Toho Gakuen or Tokyo University of the Arts, Tokyo, Japan), and the remaining two were amateur pianists who had received piano training for more than 18 years.

This experiment was approved by the University of Electro-Communications Institutional Review Board for Human Subjects Research, and was in accordance with the ethical standards in the Declaration of Helsinki. We obtained written informed consent from all participants. They were paid 3000–5000 Japanese Yen (about 30–50 US dollars, depending on their length of experience) for their participation.

### Apparatus

The experiment was performed in a soundproof room (area 4.1 m^2^, height 2.2 m, Science NAS·AL, Kawai Acoustic System, Tokyo, Japan) located in the university laboratory. The participants played a “hybrid piano” (AvantGrand N2, Yamaha, Hamamatsu, Japan), which generates sounds electronically but has the same mechanical key action as an acoustic grand piano. All participants had no prior experience of playing the hybrid piano, but they immediately adapted to it. Participants' breathing was monitored using a CO_2_ (carbon dioxide) partial pressure sensor (i.e., capnography): Their expiration was detected using a CO_2_ sensor (TG-920P, Nihon Koden, Tokyo, Japan), and its output was transduced to an electric signal via a monitoring system (OLG-2800, Nihon Koden, Tokyo, Japan). This signal was collected by a PC (operated with Window XP OS) through a 24-bit AD converter system (DF-4B32-133, Pavec Electronics Development, Tokyo, Japan) with a sampling rate of 8 KHz. Sound signals from the electronic piano was also collected using the same AD converter system. The key-pressing sequence was captured by another PC (operated with Windows 7 OS) through a MIDI interface (UA-1010, Roland, Hamamatsu, Japan). In the main experimental session, only the participants were in the soundproof room, and all experimental sessions were recorded by a video camera (EX-ZR300, Casio, Tokyo, Japan).

Most methods for monitoring respiration do not monitor the airflow itself but other correlated variables, such as the movement of the thoracic chest, the change in CO_2_ partial pressure or temperature around the mouth and nose. Only spirometer, which measures the volume of inspired and expired air, can monitor the air volume exchanged by the respiration. To use this system, however, participants have to hold a tube in their mouth or to wear a gas mask, which causes considerable stress on them. Actually, one of the authors has an experience of playing the piano with wearing a gas mask and realizes that natural breathing is difficult with this equipment; it is almost impossible to concentrate on the musical performance. In the present study, we put the highest priority on preventing the pianists from being disturbed by the respiratory measurement. In this light, the capnography is the best available method for this purpose. The nasal adaptor of the PCO_2_ sensor is small and lightweight, and is almost unnoticeable to the pianists once they start the performance. The authors confirmed this by themselves and none of the participants cared at all about the adaptor.

### Target pieces: music selected for the investigation

We chose 10 classical piano works for the experiment (Table 1), trying to cover various types of rhythm, meter, texture, and musical era. These works were all familiar to the pianists, and all participants reported that they knew these pieces even if they had no experience of playing them by themselves. Because we asked the participants to repeat these pieces six times within a limited experimental time, we extracted only parts of these pieces so that their length fit within 20–60 s, except for Debussy's work (~150 s).

**Table 1:** **Music used in the experiment**.

	**Title**
A	Scale (both hands, C major, 4 octaves, 4 repetitions)
B	Hanon, Exercise No. 7 from *Le Pianiste virtuose* (no repetition)
C	Bach, Prelude in C Major, BWV 846, 19 bars
D	Bach, Invention in C Major, BWV 772, 14 bars
E	Mozart, Theme section of Piano Sonata No. 11, K.331 1st movement (no repetition)
F	Mozart, Piano Sonata No. 16, K.545, 2nd movement, 16 bars (no repetition)
G	Beethoven, Piano Sonata No. 8, op. 13 (“Sonata Pathétique”) 2nd movement 16 bars
H	Chopin, Polonaise No. 3 in A Major, op. 40-1, (“Military Polonaise”) 8 bars (no repetition)
I	Weber, The dance section of *Aufforderung zum Tanze*, op. 65, 32 bars (no repetition)
J	Debussy, *Clair de lune, Suite Bergamasque* No. 3, 34 bars

Scores of the target pieces were all obtained from the website of the International Music Score Library Project (http://imslp.org), except for the scale (which was written by the experimenter using Musecore software [https://musescore.org/]), and were provided to the participants in advance of the experimental day.

### Procedure

A few weeks prior to the experiment day, we sent the scores to the participants, together with general instructions about the experiment and a short questionnaire asking about their history of music education. We asked the participants to practice/rehearse the pieces so that they could play them freely in the experimental session. This pre-experimental exercise was not heavy for the participants because these pieces are familiar to the pianists and do not require difficult techniques.

In the experimental session, the participants were first equipped with the nasal adaptor (YG-121T, Nihon Koden, Tokyo, Japan) of the CO_2_ sensor on their mouth and nose. The signal wires from the sensor passed behind the ear and went down along the back of the participants. Because the sensor and adaptor was small and lightweight and the wiring did not disturb the body movement, the participants' piano performance was hardly interfered with by this equipment. After the experimenter confirmed that the CO_2_ monitoring system worked, the participants were requested to sit still on a piano chair for 200 s. Here, the participants were told not to do special breathing for relaxation (e.g., deep breathing), but to just sit still and quietly for a while because the breath frequency during this period was used to estimate the breath frequency in the non-playing condition (i.e., control condition).

Next, the participants were given a several-minutes warm-up time. They could freely play the piano; they could either acclimate themselves to the instrument, do finger exercises, or practice the target pieces. They could continue practice until they felt ready to start the performance. From this time, we mentioned nothing about breathing so as to prevent the participants from paying unnecessary attention to their breathing.

After the warmup, the main experiment started. The pianists were asked to play an excerpt six times consecutively in each block. Within each block, they could voluntarily start a trial, but they were requested to re-start the next trial with a fresh mind, after a several-second pause. Only the participant was in the soundproof room in the experimental block, and the start and end of each block was instructed by the experimenter from the outside (thus, the pianists did not have to count the trials so that they could concentrate on their performance). The experimenter let the player play one or two more times when he noticed an operational error or a mistake by the player during the experiment. Participants were allowed to see the score in the session. They were encouraged to play the piano seriously and in a relaxed fashion as in the way they would usually practice at home, and not nervously as in examinations and recitals. They were told to play through the excerpt even if they made some mistakes. In fact, all participants successfully performed all pieces without stopping (several participants made mistakes on the number of repetitions in scale playing), and data from almost all trials could be used as valid experimental data. After the end of the block, the experimenter opened the door of the soundproof room, and prepared for the next excerpt. The participants were allowed to take a rest between the blocks.

All participants played the excerpts in the fixed order shown in the list in Table 1. The playing tempo was specified at 80 bpm (beats per minute) for Excerpts A and B, by means of a metronome. Note that this tempo was rather slow for skilled pianists because students in music school are often required to play scales (Excerpt A) at 130–140 bpm in an examination. As for the other excerpts, the tempo was not specified so that the participants could play the piano as they wished.

The total time of an experimental session was ~90 min. After the end of the session, the participants were asked to fill in a brief questionnaire, asking (1) whether (and when) they were aware of breathing in their usual piano performance, (2) whether (and when) they consciously tried to control the breath timing during the usual performance, (3) whether (and how) they had been instructed about the breathing in piano performance by their teachers, (4) whether they cared about the breath timings of other players in ensemble performance, and (5) whether they tried to match the breath timing with the other players in ensemble performance.

### Signal processing

**Figure 1:**
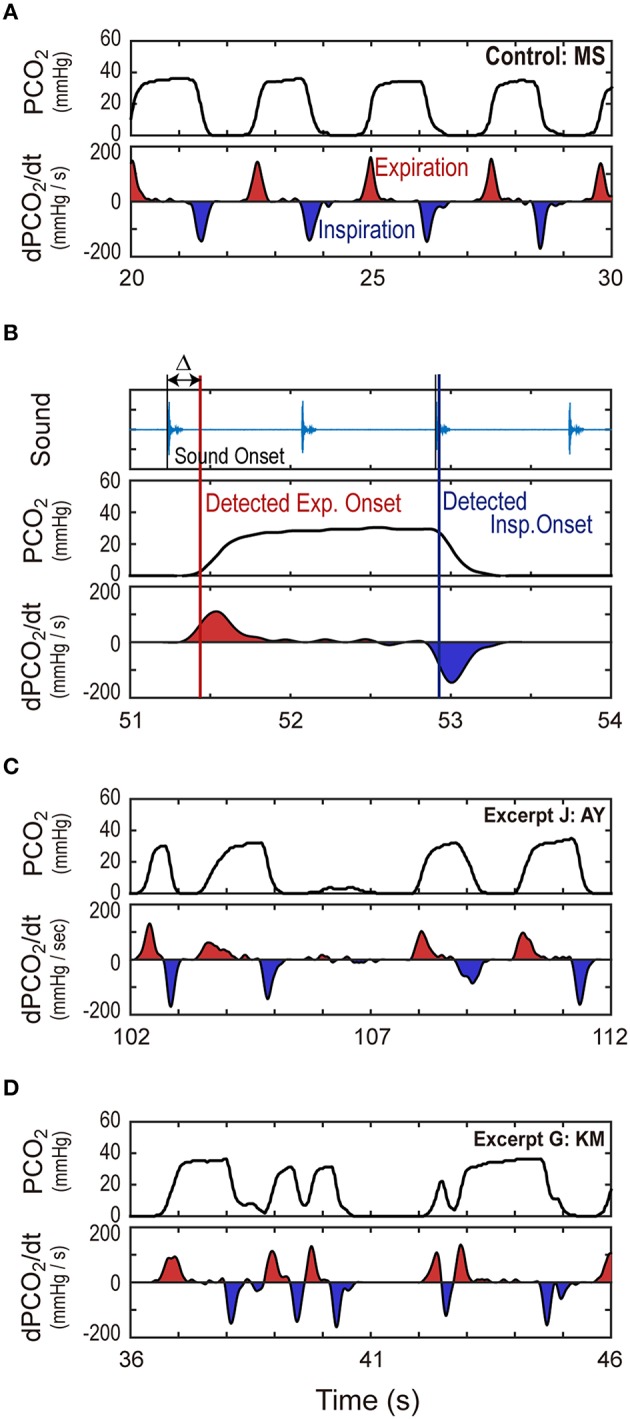
**Typical behavior of CO_**2**_ partial pressure. (A)** A temporal profile of the CO_2_ partial pressure (PCO_2_) of a participant is shown, together with its temporal derivatives. **(B)** A PCO_2_ pattern is shown when an experimenter inspired and expired for every two beats of metronome sounds whose signal is depicted in the top panel. Vertical red and blue lines were the timing of the expiration and inspiration detected by the signal processing program. Although little difference was found between the detected inspiration onset and the sound onset, there was non-negligible time difference (Δ ~ 200 ms) between the detected expiration onset and the sound onset. **(C)** An example of temporally irregular PCO_2_ patterns is shown. In this example, the amplitude of PCO_2_temporal derivative greatly varied among individual breaths. **(D)** Another example of irregular PCO_2_ patterns is shown. In this example, PCO_2_ started to increase before it reached the zero level.

Signal processing was performed with Matlab software and its toolboxes (Mathworks, Natick, MA, USA). We applied several pre-processing steps to the breath signal (i.e., CO_2_ partial pressure: PCO_2_). Because the sensor's output signal contained specific pulses for automatic calibration (every 10 min), we first removed these pulses (we lost some breath data because of these pulses, but it was only a small portion), together with translating the electronic signal to the partial pressure value (using this calibration signal). Next, we applied a low-pass filter (4th order Butterworth filter, cut-off frequency 20 Hz) to smooth the signal. Moreover, we applied numerical differentiation to PCO_2_ to readily distinguish the direction of respiration (i.e., inspiration vs. expiration; See Figure 1 for example).

Sound signals were used to monitor the temporal position on the score and to align the data from the different trials. To align the temporal axes of the different trials, we performed the following processing. First, we performed frequency analysis using a windowed FFT method, where the window width was 4096 points (512 ms) and the step of window shift was 512 points (64 ms). Next, applying the “dynamic time warping” or “DP matching” technique (Sakoe and Chiba, 1978) to the obtained sound spectrogram, we found the correspondence between the sound signals of different trials for every window step (64 ms). We confirmed that this matching process worked normally by checking that the time axes of the corresponding trials were continuously matched to each other. Then, making use of this matching information, we mapped the sound signals, breath patterns, and keying sequences of multiple trials onto a standard time axis, that is, that of the final trial. The average breath pattern, which we will explain below, was calculated on this standard time scale.

Because the breath signal simply represents the CO_2_ partial pressure around the participant's mouth and nose, we cannot use it to determine the respiratory air flow; what we can see is the increase and decrease in PCO_2_, that is, the switch between inspiration and expiration. Thus, our discussion is basically focused onto the timing of the breathing. Figure 1 shows some typical patterns of the temporal change in PCO_2_ and their temporal derivative. As shown in Figure 1A, generally, PCO_2_ started to increase when the participant blew out a breath (i.e., expiration) and remained at a high level for a while. Then, it decreased to zero as the participants drew fresh air from the surroundings (i.e., inspiration) and stayed at a zero level for a while, and then increased again. Because of the saturation of gas concentration, however, it is hard to discriminate whether a participant stops breathing or continues to exhale/inhale when PCO_2_ remains at a high level or at a zero level.

Under this condition, it was technically easy to detect the onset of expiration because the increase in PCO_2_ was generally drastic. Thus, we used the onset timing of expiration as the primary feature of breathing. To be more specific, we detected the “expiration onset” as the time when PCO_2_ exceeded 5% of the maximal PCO_2_ (within the trial) from the zero level. Breath interval was defined as the interval between two consecutive onsets of expiration, and breath frequency was defined as the inverse of the breath interval.

However, we should note that a non-negligible dissociation exists between the actual air flow of breathing and the change in sensed PCO_2_ value. It takes some time until the CO_2_ gas produced in the lung reaches the sensor in front of the mouth and nose through the trachea and oral cavity. Figure 1B shows the breath timing when an experimenter breathed in response to the sound of the metronome at 72 bpm (one respiration cycle every four beats, i.e., 18 breaths in 1 min). As can be seen in the figure, PCO_2_ onset was ~200 ms behind sound onset. When we determine the PCO_2_ onset as the time when PCO_2_ exceeded 5% of its maximal value, the histogram of the discrepancy between the two onsets (Δ in the figure) had a steep peak at 200 ms. The size of the discrepancy was the same for another experimenter, and moreover, the same discrepancy was detected when the experimenters actively made key strokes in accordance with the expiration. Thus, we estimated the actual breath onset as about 200 ms before the detected PCO_2_ onset. Although this discrepancy has no effect on the breath interval and frequency, we need to take account of this point when examining the temporal correspondence between musical events and respiration. In particular, the temporal profiles of PCO_2_ might possibly give a wrong impression on the actual respiration timing. Thus, below, we will explicitly indicate the estimated onsets of the expiration. Related to this point, we should note that this discrepancy was not observed for the inspiration: we can see no time difference between the sound onset and the start of the PCO_2_ reduction (See Figure 1B).

Moreover, we must point out that the temporal pattern of PCO_2_ in piano playing showed not only regular back-and-forth patterns (as in Figure 1A), but also rather complicated behavior. For example, the amplitude of the temporal derivative of PCO_2_ (i.e., the slope of the temporal change of PCO_2_) greatly varied among individual breaths. In Figure 1C, the slopes of the increases/decreases of PCO_2_ differed across different breaths, which results in the different amplitudes of the temporal derivative (dPCO_2_/dt). This is presumably because the air flow of respiration was not constant (i.e., greater air flow would bring steeper change in PCO_2_). Moreover, PCO_2_ often started to decrease before it reached the saturated level (around 106 s in Figure 1C, and around 42 s in Figure 1D), and started to increase before it reached the zero level (around 40 and 43 s in Figure 1D). Taking account of these facts, we also regarded the beginning of the fast expiration as expiration onset even if it did not start from the zero level. More specifically, we treated the events satisfying the following conditions as expiration onsets: (1) the temporal derivative of PCO_2_ exceeded 100 mmHg/s, (2) time to the next inspiration was longer than 300 ms, and (3) change in PCO_2_ until the next inspiration was larger than 20 mmHg. We checked all events manually and adjusted the irregular cases (at most a few times within a trial).

Because the raw temporal pattern of PCO_2_ contains richer information than the timing of inspiration/expiration onsets, we will refer not only to parametric variables, such as breath interval and its variation, but also PCO_2_ patterns themselves to examine the characteristics of breathing. For example, we calculated the “average breath pattern” by calculating the mean of PCO_2_ of every time count of the different trials after matching their temporal axes. The average breath pattern [*avePCO*_2_(*t*)] is defined mathematically as follows:

$$\begin{array}{l} \text{ave}PCO_{2}\left(t\right)=\frac{1}{N_{trial}}\overset{N_{trial}}{\underset{k=1}{\sum }}PCO_{2}\left(t;k\right), \end{array}$$

where *PCO*_2_(*t*; *k*) is the PCO_2_ value at regularized time *t* of the *k*th trial and *N*_trial_ is the number of the trials. This average pattern tells us the inter-trial consistency of the breath pattern. If it shows a distinct profile, then the participant breathed in a consistent manner over the repeated trials. Contrastingly, a flat average pattern implies that the breath pattern varied trial by trial.

As for the control condition, we manually examined the details of the breath pattern of each participant and extracted the time region over which the breath pattern was stable. Then, we detected the expiration onsets from the extracted region, and calculated the breath interval in the control condition.

## Result

### Data acquisition

We successfully collected sound and PCO_2_ signals from all participants though we lost data from 29 trials because of players' mistakes and the experimenter's operational error. In total, data from 889 trials were used for further analysis.

### Performance time

Table 2 summarizes the performance time of all excerpts. Playing times differed considerably between the participants: the difference between the fastest and slowest players reached around 30 s for Excerpts C and F, and 55 s for Excerpt J. However, standard deviation (within participant) was generally around 0–2 s for most participants (except for excerpt J which had a standard deviation of 2–5 s), meaning that every participant repeated the trials with a consistent speed. In order to confirm that the participants played each excerpt in a consistent speed and that the within-participant variability was smaller than the between-participant variability, we calculated the intra-class correlation coefficient ICC(1, 1). Resultantly, the values were 0.45 (A), 0.39 (B), 0.97 (C), 0.98 (D), 0.97 (E), 0.98 (F), 0.88 (G), 0.93 (H), 0.99 (I), and 0.99 (J); they were close to 1 (*p* < 0.001) except for A and B, meaning that the inter-trial (i.e., within-pianist) variability was significantly smaller than the inter-pianist variability. It is natural that the values were smaller for Excerpts A and B because they were played with metronome.

**Table 2:** **Summary of performance time**.

	**Mean (SD) (s)**		**Min/Max (s)**	
A	43.6	(0.4)	43.0	44.2
B	43.9	(0.3)	43.6	44.4
C	63.3	(7.6)	46.5	76.5
D	34.5	(3.2)	28.2	41.6
E	55.0	(5.7)	42.0	62.0
F	49.5	(7.8)	36.4	64.4
G	56.9	(3.8)	51.1	65.6
H	18.7	(1.2)	17.4	21.8
I	26.7	(5.9)	20.2	42.5
J	131.3	(13.9)	105.7	161.2

### Mean breath interval

**Figure 2:**
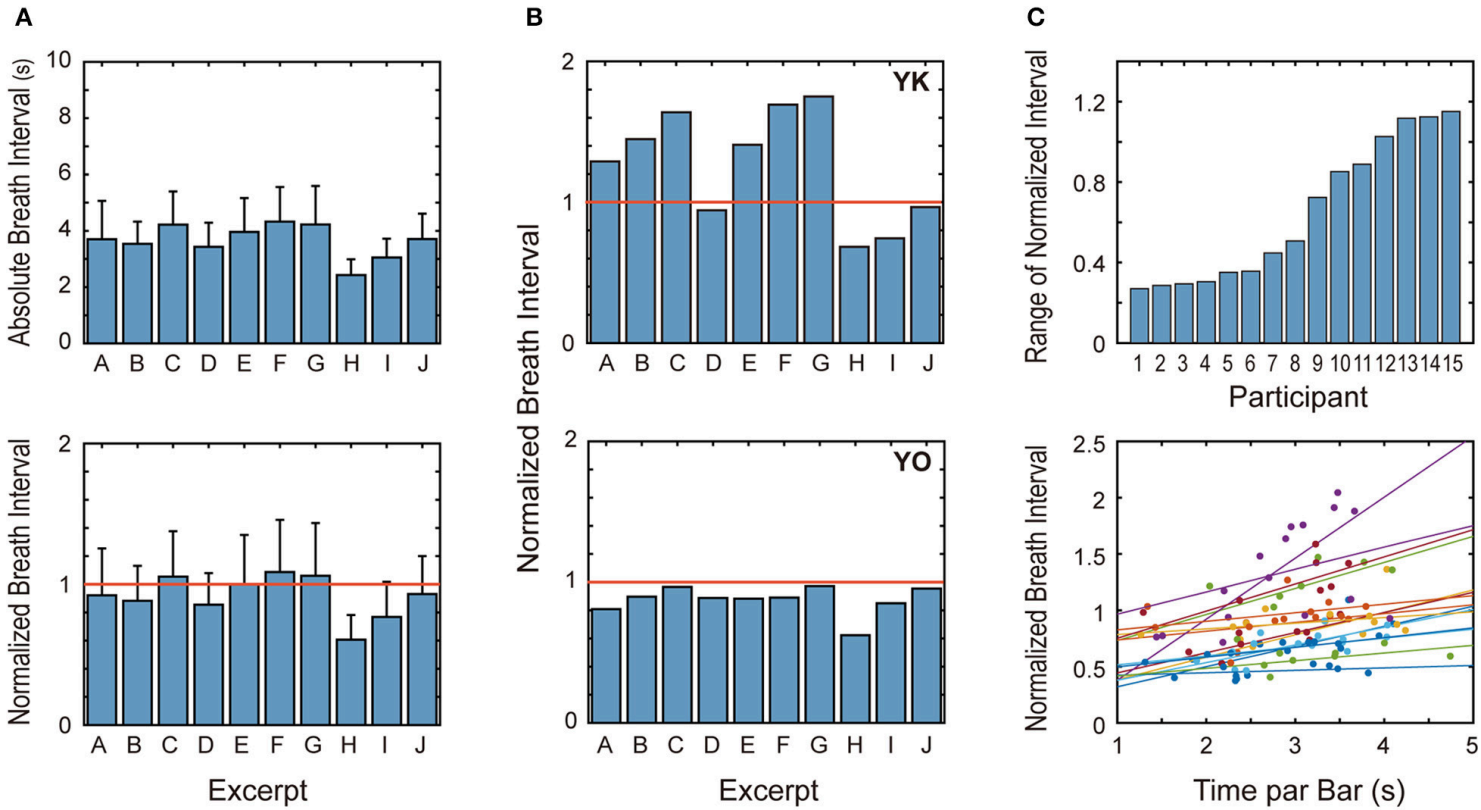
**Mean breath interval of different excerpts. (A)** Inter-participant means of absolute breath intervals and normalized ones for different music excerpts are summarized. Breath intervals were significantly different between the excerpts. **(B)** Average breath intervals of two specific participants are shown. **(C)** The upper panel shows the difference between the maximum and minimum values of normalized breath intervals for 15 participants in a sorted manner. The lower panel is a scatter plot between “time per bar” and “normalized breath interval” for all participants. Each color represents one participant and the line having the same color is its regression line. All regression lines have positive slopes, indicating that the breath intervals became longer for the excerpts played in slower tempo.

We examined the mean breath interval (to be exact, the mean expiration interval) of each excerpt. Figure 2A summarizes the data for the absolute breath intervals and for the normalized ones (i.e., mean interval in every music condition divided by that in the control condition). Both the absolute and normalized intervals apparently differed between the excerpts. The result of a within-subject ANOVA detected a significant effect of the excerpt on the absolute intervals [*F*_(9, 124)_ = 10.6, *p* < 0.001], and on the normalized intervals [*F*_(9, 124)_ = 10.8, *p* < 0.001].

Looking at this figure more closely, we can see two apparent tendencies. First, the breath interval was notably shorter for Excerpts H (Chopin) and I (Weber). *Post-hoc* multiple-comparison (with Bonferroni adjustment) showed that breath interval for Excerpt H was significantly shorter than the remaining excerpts (except for Excerpt I) and that for Excerpt I was significantly shorter than Excerpts C, E, F, and G. Because pianists need to repeat fast and intense key strokes with many fingers for Excerpt H, some may imagine that powerful key stroke accelerates breathing. Considering that this short breath interval was observed from the very beginning of the performance and that Excerpt I does not require intense key strokes, however, we should think that the musical tempo may be a more important factor determining the breath interval.

Second, the normalized breath interval was greater than one for some excerpts (i.e., C, E, F, and G), meaning that the breath interval in piano playing can be longer than in quiet sitting. Figure 2A shows only average data, but some participants exhibited much slower breathing compared with the control condition, as in the upper panel of Figure 2B. At least for these participants, breath intervals were lengthened by some factors, compared to the resting condition.

Though the statistical test showed a significant effect of the musical work on the breath interval, some participants showed only slight difference between the musical works (lower panel of Figure 2B). We quantified the variation of breath interval between different excerpts by the difference between maximum and minimum values of normalized breath intervals. The upper panel of Figure 2C shows this measure of each participant in a ranked manner. This measure ranged from 0.2 to 1.2, indicating that the effect of musical works on the breath interval depended on the individual pianists.

Finally, to obtain further evidence on the relationship between tempo and breathing rate, we examined the correlation between the playing speed and mean breath interval. The lower panel of Figure 2C shows a scatter plot of “time per bar” and “normalized breath interval” for all participants. All of the regression lines have positive slopes. In order to test the effect of tempo on the normalized interval, we used the following linear-mixed model (LMM):

$$\begin{array}{l} \text{normalized interval}\sim \text{tempo }+\;(\text{tempo }|\text{ participant})\\ \;(\text{by Wilkinson notation}), \end{array}$$

which deals with tempo as the fixed effect and participant as the random effect. The estimated slope of the fixed effect was 1.13, *t*_(116)_ = 4.45, *p* < 0.001, confirming that the positive correlation was detected for the grouped data; in other words, the respiration was faster when playing music faster.

### Fluctuation of breath interval within excerpt

**Figure 3:**
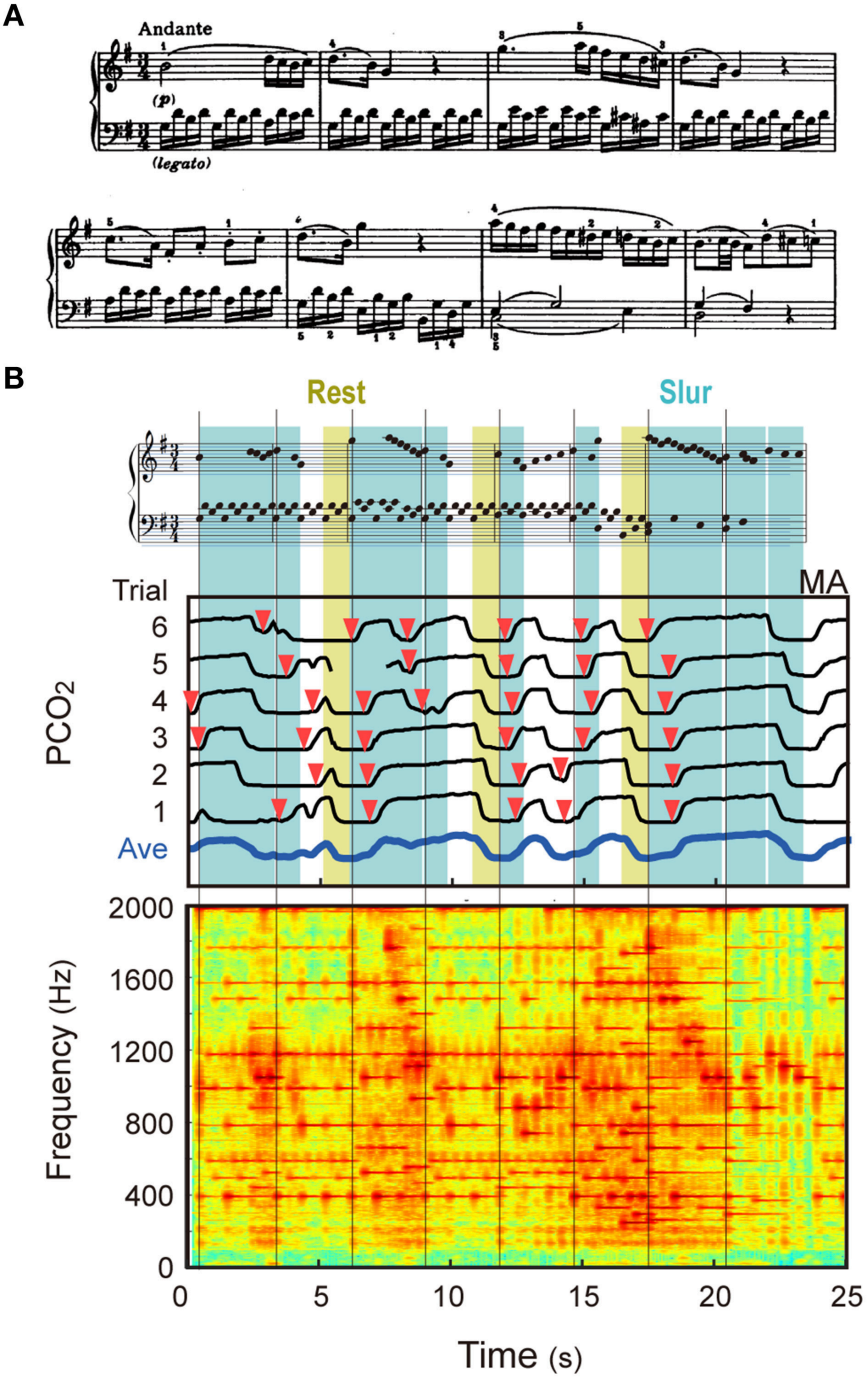
**Example of breathing pattern in piano playing. (A)** The score of the first 8 bars of Excerpt F used in the experiment is shown. **(B)** The middle panel shows the PCO_2_ temporal patterns of six trials of one Participant, together with their averaged pattern [*avePCO*_2_(*t*)]. The temporal axes for these patterns were matched to each other using the dynamic time warping method. The bottom panel shows a part of the sound spectrogram used for the time warping. The top panel shows the rearranged score so that the timing of each note is arranged in accordance with the physical time. Each vertical line indicates the timing of the first note of each bar, and red arrows represent the timings of the expiration onsets estimated from the PCO_2_ patterns. The respiratory pattern of this participant apparently corresponds to the musical events.

Figure 3 depicts PCO_2_ patterns of six repetitions of Excerpt F (Mozart's Sonata) of one participant, together with their averaged pattern [i.e., *avePCO*_2_(*t*)]. It also shows the sound spectrogram and a regularized score in which the note positions were arranged according to the physical time (together with the original score for reference). Each vertical line indicates the timing of the first note of a bar, and red arrows represent the timings of the expiration onsets estimated from the PCO_2_ patterns (See Signal Processing).

Three facts can be observed from this result. First, the breath interval was never uniform, but varied dynamically within a short duration (i.e., 8 bars or 24 s). This suggests that only mean breath interval over a long period is not enough to discuss the nature of breathing in piano playing. Second, the PCO_2_ patterns in different trials resemble each other [as a result, *avePCO*_2_(*t*) shows a well-modulated profile], meaning that breath timing was consistent for every performance for this participant. This inter-trial consistency of breathing patterns will be discussed in the next section. Third, the breath timing generally corresponded to the musical events. Specifically, this participant inhaled around the rest notes (indicated by the yellowish bands), and exhaled about 1 s after the beginning of new melodies. In addition, she made few breaths within the slurred parts. These features will be further examined below.

**Figure 4:**
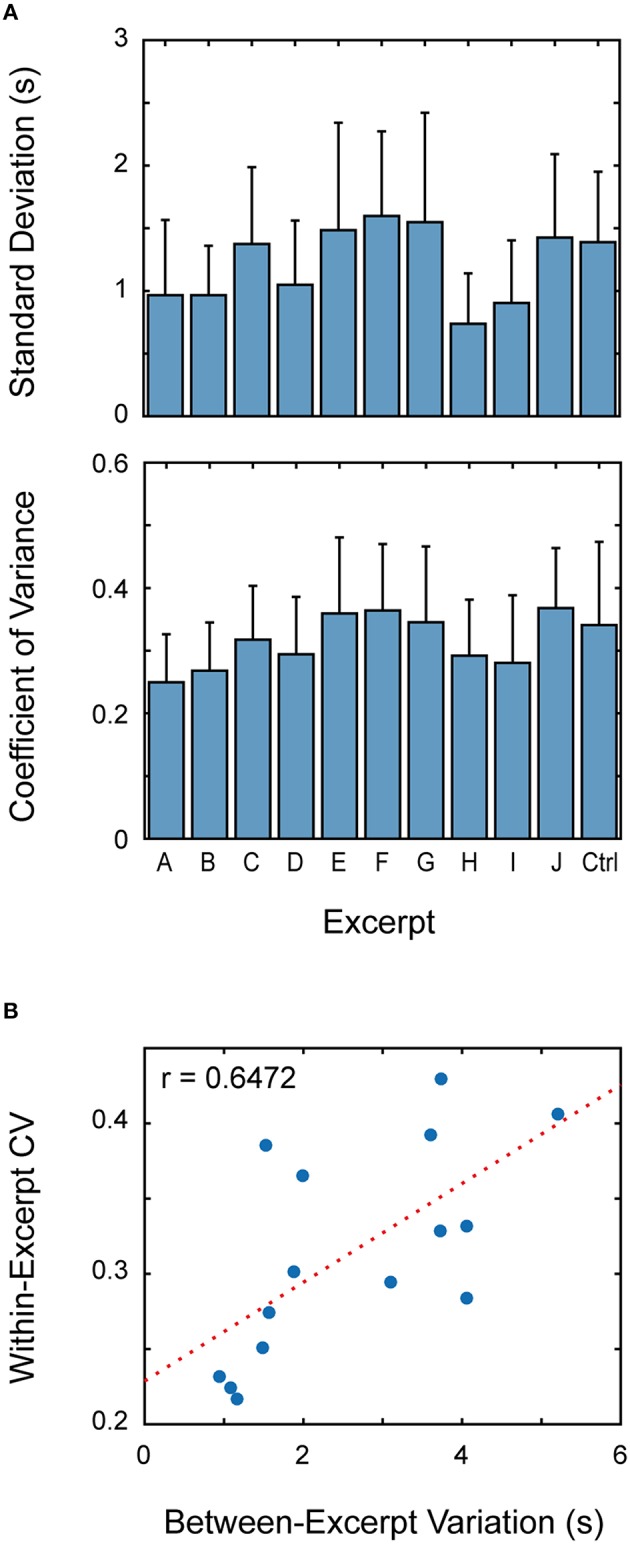
**Fluctuation of expiration intervals. (A)** The upper panel shows the inter-participant averages of standard deviation of breath intervals within music excerpts, and the lower panel shows those of coefficient of variation (CV). Both standard deviation and CV value significant differed between the excerpts. **(B)** The relationship between the variation of breath interval between different music pieces and that CV value within music pieces is plotted. There is positive correlation between these variables.

In order to see the statistical property of the fluctuation of breath interval, we calculated the variability of breath intervals for each excerpt and for the control condition. Figure 4A shows inter-participant averages of the standard deviation of breath intervals and those of coefficient of variation (CV: standard deviation/mean). Both standard deviation and CV value differed between the excerpts. Apparently, they took smaller values for the pieces for finger exercise (i.e., Excerpts A and B) and those with faster tempi (i.e., Excerpts H and I). Note that variability in the resting condition was not the smallest.

We confirmed the difference in variability by statistical tests. When we applied a Levene's test, a method of testing for the equality of variance, separately for individual participants, first, a significant difference was detected between the excerpts for all participants. The Miller-Feltz test (Miller and Feltz, 1997), a method of testing for homogeneity of CV, detected a significant difference for 13 out of 15 participants. Next, we examined the effect of excerpt on standard deviation and CV values using the within-subject ANOVA. The effect was significant both for standard deviation [*F*_(10, 138)_ = 6.24, *p* < 0.001] and for CV [*F*_(10, 138)_ = 3.94, *p* < 0.001]. *Post-hoc* multiple-comparison with Bonferroni adjustment detected significant differences between the pairs (H, E), (H, F), (H, G), (H, J), and (I, F) for standard deviation and the pair (A, J) for CV.

Here, we should note that the variability of breath interval was also different between the participants. The inter-excerpt averages of CV values (for different pianists) ranged from 0.9 to 5.4, and their mean was 2.6. Here, some may suspect that participants whose breath intervals were variable within a music piece also showed a large variance between different musical pieces. To examine this conjecture, we plotted the relationship between the mean CV values (i.e., within-excerpt variance) and the range of normalized breath interval (i.e., between-excerpt variation, See Figure 2C) on a 2D plane (Figure 4B), where each circle represents one participant. Rough but significant correlation can be seen between these two measures (*r* = 0.6472, *p* < 0.01), supporting the conjecture.

### Inter-trial consistency of breath pattern

In the previous section, we showed an example that a participant breathed at similar timings for every performance (Figure 3). We further discuss this point in this section.

In Signal Processing, we pointed out that the behavior of average PCO_2_ pattern [*avePCO*_2_(*t*)] can be an indicator of the inter-trial consistency: greater amplitude of *avePCO*_2_(*t*) means higher consistency between different trials. In order to quantify the inter-trial consistency, we calculated standard deviation of the averaged PCO_2_ (referred to as “SDAP” below) for every combination of the excerpts and participants. Mathematically, SDAP is defined by,

$$\begin{array}{ccc} \bar{avePCO_{2}} & = & \frac{1}{N_{sample}}\overset{N_{sample}}{\underset{t=1}{\sum }}avePCO_{2}\left(t\right)\\ \text{SDAP} & = & \sqrt{\frac{1}{N_{sample}}\overset{N_{sample}}{\underset{t=1}{\sum }}{\left(avePCO_{2}\left(t\right)-\bar{avePCO_{2}}\right)}^{2}} \end{array}$$

where *N*_sample_ is the number of sample times. A larger value of SDAP indicates higher inter-trial consistency because greater amplitude of *avePCO*_2_(*t*) brings larger standard deviation.

**Figure 5:**
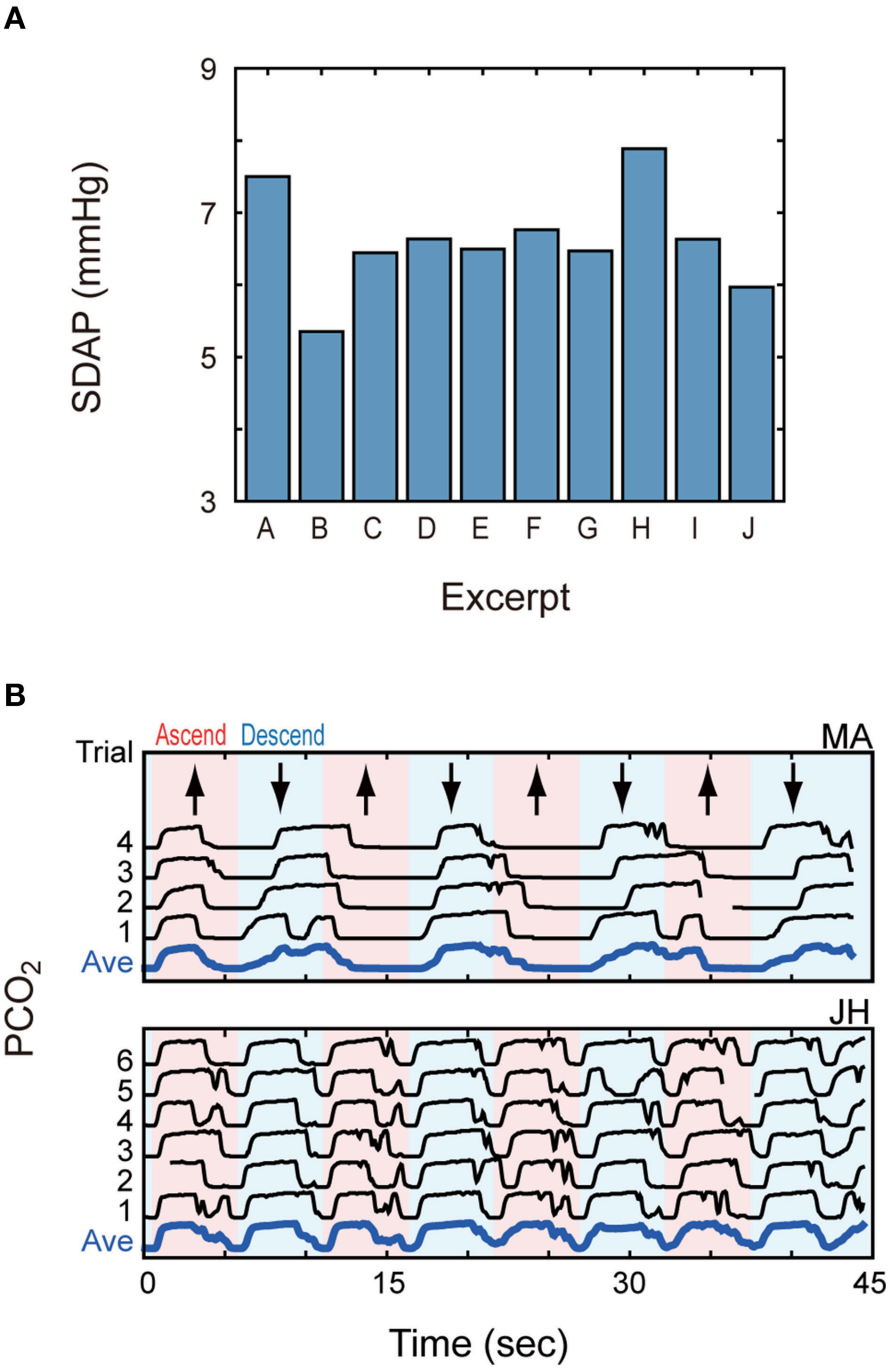
**Inter-trial consistency of breath pattern. (A)** Standard deviation of inter-trial average PCO_2_ values (SDAP) is plotted for every music excerpt. **(B)** PCO_2_ patterns of two participants for Excerpts A (scale) are shown. The pink and blue backgrounds indicate upward and downward sequences, respectively. These participants exhibited quite consistent and structured breath patterns for this monotone piece.

Figure 5A summarizes the inter-participant average of SDAP values for every music piece. SDAP took smaller values (i.e., lower inter-trial consistency) for Excerpts B (Hanon) and J (Debussy), and larger values (i.e., higher inter-trial consistency) for Excerpts A (scale) and H (Chopin). These observations were supported by statistical tests. A within-subject ANOVA detected a significant effect of the excerpt, *F*_(9, 126)_ = 4.21, *p* < 0.001. *Post-hoc* multi-comparison with Bonferroni adjustment detected significant differences between the pairs of (A, B), (B, H), and (H, J). We also analyzed the result using a LMM to test the effect of the pianists (i.e., individual difference). We prepared the following LMM models and compared them by the likelihood ratio test:

$$\begin{array}{l} \text{Model 1}:\text{SDAP}\sim \text{excerpt }+\;(\text{1 }|\text{ participant})\;(\text{with the random}\\ \text{ effect of participant}),\text{ and}\\ \text{Model 2}:\text{SDAP}\sim \text{excerpt }(\text{without the random effect}). \end{array}$$

Model 1 was significantly better than Model 2 [$\chi_{\left(1\right)}^{2}=20.575$, *p* < 0.001], indicating that there was a significant effect of participants. Note that both models showed significant effects of the fixed-effect coefficients [*F*_(9, 140)_ = 4.512, *p* < 0.001 for Model 1, and *F*_(9, 140)_ = 3.403, *p* < 0.001 for Model 2], supporting the effect of musical piece.

This analysis led us to find contrasting results between two finger exercise pieces, Excerpts A and B. If the breathing is coupled with musical organization, systematic breathing would not be observed in those pieces that have no clear musical structure (such as finger exercises) and eventually, inter-trial consistency would be degraded. This was the case for Excerpt B, but not for Excerpt A. Indeed, some participants showed quite consistent respiratory patterns for Excerpt A. As shown in Figure 5B, one participant (MA) regularly inspired in the ascending sequence and expired in the descending sequence, and another participant (JH) had one breath cycle (inspiration and expiration) for either ascending or descending sequence. Some other participants exhibited a pattern synchronized with smaller units (e.g., octave units). Such structured breath patterns were found in 6 out of 15 participants, which brought a larger SDAP in the above analysis. Therefore, even if a musical work in the score looks monotonous and musically colorless, the breathing pattern can be correlated to some “organization” of the piece.

### Relationship to musical events and organization

In Figure 3, we have shown an example that the breath timing appeared to be related to the musical events. In this section, we examined this point focusing on some specific musical events and structures.

#### Music onset

**Figure 6:**
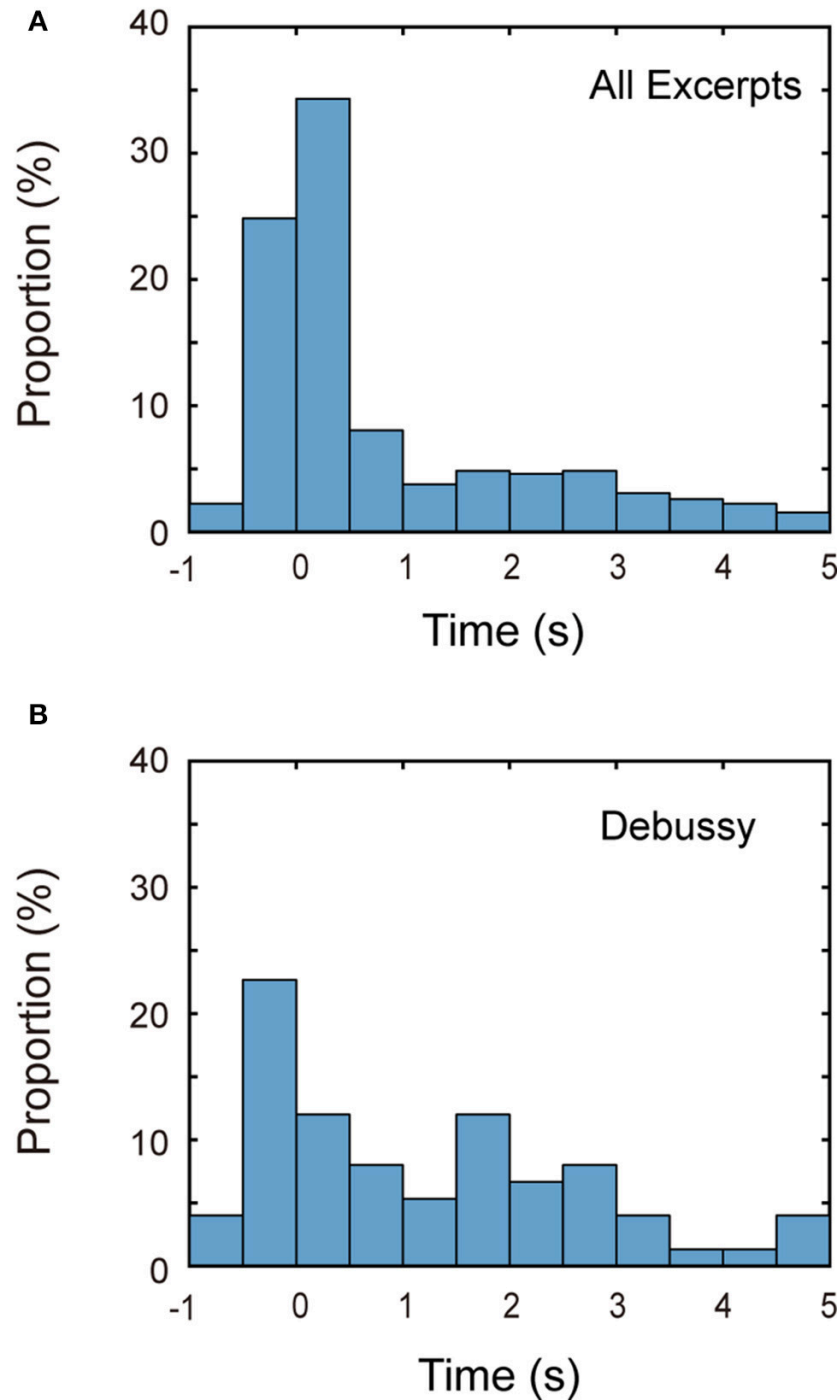
**Distribution of first expiration time. (A)** Histogram of the timing of the first expiration after 500 ms before the music onset is plotted. The first expiration is concentrated on just after the music onset. **(B)** Histogram for Excerpt J is shown. The distribution is apparently broader than the other musical pieces.

We first examined the timing of the breathing at the music onset. Figure 6A shows the histogram of the first expiration time after 500 ms before the sound onset. Apparently, the first expiration was concentrated around the music onset: over 66% of the entire trials, the first expiration was done by 1 s after the onset. This first expiration ratio (66%) was significantly higher than the expected ratio [37.5% = 1.5 s/4 s, *t*_(888)_ = 17.57, *p* < 0.001]. This feature was commonly observed for almost all excerpts and participants, implying that the breath timing is tightly coupled with the music onset.

As for one exceptional case, the distribution of the first expiration timing was apparently broader for Excerpt J (Debussy), as shown in Figure 6B. Actually, the ratio of first expiration by 1 s after the music onset was only 41%. The result of a within-subject ANOVA showed that the ratio was significantly different between the excerpts, *F*_(9, 125)_ = 4.10, *p* < 0.001. *Post-hoc* multiple-comparison detected significant differences between the pairs (A, J), (B, J), (H, J), and (I, J). We have pointed out that SDAP took a small value (i.e., low inter-trial consistency) for this excerpt. Though we do not go into more detail, this excerpt seemingly brought somewhat peculiar breathing characteristics.

On the other hand, two participants showed broader distribution. Especially, median of the first expiration time of one participant was 1.5 s, much greater than the other participants (0–0.6 s). Actually, her PCO_2_ level often remained at zero for a few seconds after the music onset (See Participant EA in **Figure 8**), suggesting that she regularly stopped breathing for a few seconds after starting music.

#### Rest

We have already shown an example that a participant often inhaled but rarely exhaled at the rest notes (Figure 3). In order to test whether this feature was observed for all participants, we examined the occurrence of inspirations and expirations at four rest notes (to be exact, within the time period corresponding to the rest notes) in the 2nd, 4th, 6th, and 12th bars of Excerpt F.

Ratios of occurrence were 40% for inspiration and 22% for expiration. The ratio for inspiration was somewhat different between the four bars (51, 33, 44, and 31% for the 2nd, 4th, 6th, and 12th bars, respectively) whilst that for expiration was almost constant (24, 22, 22, and 18%). On the other hand, there was considerable difference among the participants: the ratios ranged 16–71% for inspiration and 0–46% for expiration.

For this excerpt, the length of time period for a rest note was about 1 s, and the mean breath interval was about 4 s (See Figure 2). If the breath occurs randomly and uniformly over the music piece, therefore, we can estimate that the expected ratio of occurrence of inspiration/expiration at the rest notes should be about 25% (= 1/4). The ratio for inspiration (= 40%) is significantly greater than this expected ratio [*t*_(86)_ = 6.190, *p* < 0.001], meaning that the pianists were likely to inhale in accordance with the rest notes, at least for this excerpt.

#### Slur

We also pointed out above that the participant made few breaths during the slur parts (Figure 3). To be more concrete, expiration occurred around the beginning of slur parts whilst inspiration hardly occurred during the slur parts. In order to quantify this observation, we examined the occurrences of inspirations and expirations during four slur parts in the 1st, 3rd, 7th, and 11th bars [which lasted over 1 meter (= 3 bars)] of Excerpt F.

Resultantly, ratios of occurrence were 54% for inspiration and 73% for expiration. The individual ratios for the four bars were 39, 54, 56, and 66% for inspiration, and 64, 85, 76, and 65% for expirations. Therefore, generally, expiration occurred more frequently than inspiration during the slur parts. Because the length of a slur was about 3 s and the mean breath interval was about 4 s, the expected ratio of inspiration/expiration occurrence is about 75% (= 3/4). The ratio of inspiration occurrence (54%) was lower than the expected ratio [*t*_(86)_ = 4.581, *p* < 0.001], suggesting that inspiration was less likely to occur during the slur parts.

Again, we found large inter-participant difference: the ratios of occurrence ranged 20–83% for inhale and 35–94% for exhale.

#### Contrapuntal piece

In the previous sections, we have shown that the pianists' respiration was related to the music onset, rests and slurs. As an attempt to examine the effect of more global musical organization, next we focused on Excerpt D (Bach's Invention), a two-part contrapuntal piece: in this musical piece, two musical voices are often played in a pairwise manner (i.e., subject and counter-subject). We analyzed whether or not such polyphonic organization was correlated with the pianists' breathing pattern.

**Figure 7:**
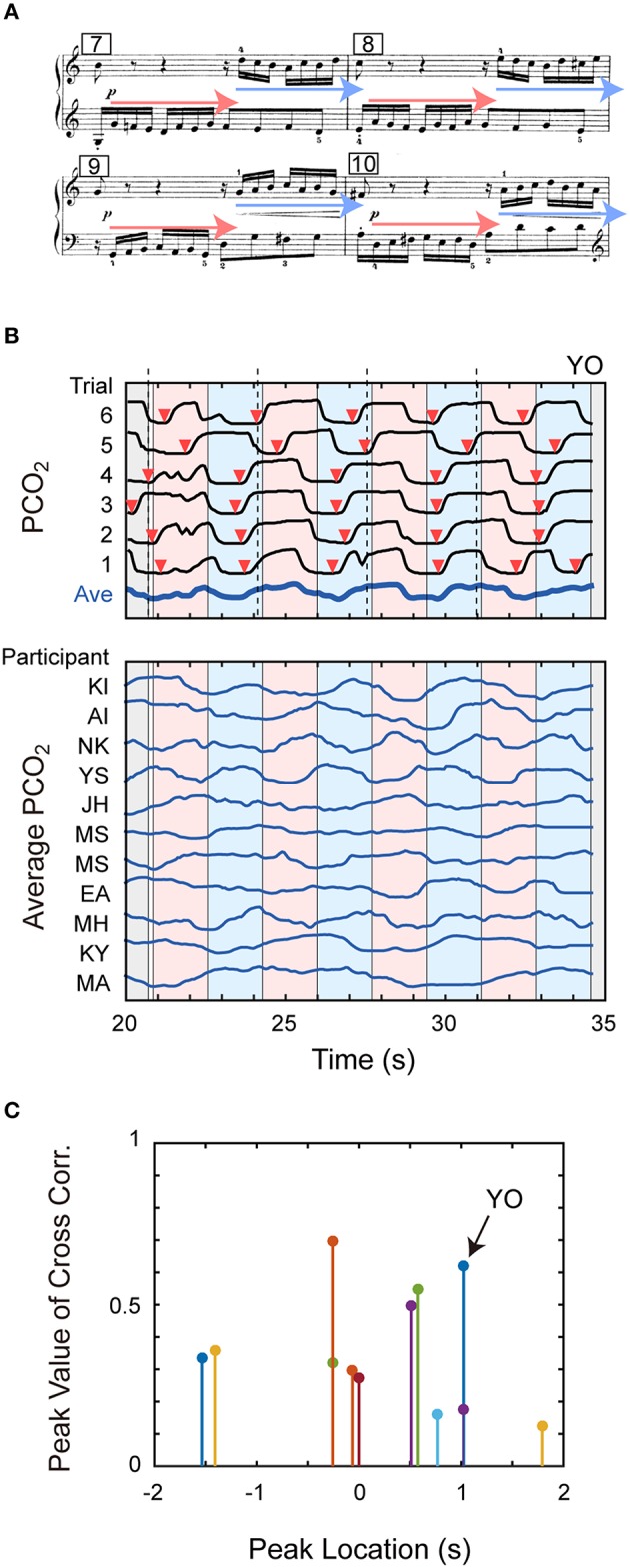
**Breath patterns for an excerpt having two voices. (A)** The score of the final part of Excerpt D is shown. As indicated by the reddish and bluish arrows, pairwise subjects are played four times in this part. **(B)** Top panel shows temporal patterns of PCO_2_ of individual trials and its average pattern for one participant. Bottom panel shows the average PCO_2_ patterns of other 11 participants along the matched time axis. Vertical broken lines indicate the timing of the first notes of the bars, and reddish and bluish bands represent the timing of subjects indicated by the reddish and bluish arrows in the score, respectively. **(C)** Temporal correlation between the breathing pattern and musical structure is depicted. This figure summarizes the locations and values of positive peak of cross correlation function of the average PCO_2_ pattern and subject vs. counter-subject structure for individual participants. See text for details.

Specifically, we focused on the part from the 7th bar to the 10th bar of this musical work, where pairwise subjects are played four times (Figure 7A). An example of PCO_2_ pattern is shown in the upper part of Figure 7B. In this figure, the vertical broken lines indicate the timing of the first note of the bars, and reddish and bluish bands represent the subjects indicated by the reddish and bluish arrows in the score, respectively. Although there were some inter-trial variations, the expiration onsets (indicated by the red arrows) were generally located within the subject played by the right hand (i.e., bluish band), or almost coincided with the onsets of the right-hand subjects. In addition, the averaged breath pattern looks correlated with the reddish-bluish pattern in the figure. This suggests the possibility that this participant expired in accordance with the right-hand subject.

The lower part of Figure 7B shows *avePCO*_2_(*t*) of other 11 participants, where the temporal axes were re-arranged between different participants, using the same method used for time mapping within the participants (we omitted data from three participants because the dynamic time-warping algorithm did not work normally for them). Looking close at individual patterns, we see some correlation between the PCO_2_ pattern and the contrapuntal structure for several participants (e.g., Participants KI, NK, and YS). Note that some other participants showed a longer structure of breathing (e.g., Participants JH and MA).

In order to quantify this observation, we defined a binary function [*B*_(*t*)_ = +1 or −1] whose value represents whether the subject was played by the right hand or by the left hand at time *t*, and calculated the cross-correlation function between this function and the average PCO_2_ pattern [i.e., *avePCO*_2_(*t*)]. Then, we extracted its positive peak value and peak location [within the time length of unit subject (= 1.7 s)] for each participant. Figure 7C summarizes the result, where the horizontal and vertical axes represent the peak location and peak value, respectively. Here, the peak value represents the magnitude of correlation and the peak location represents the breath timing relative to polyphonic structure. If the expiration onset matches the start of right-hand subject, the peak location would be 0 s (it would be +1.7 or −1.7 s if the expiration onset matches the start of left-hand subject). For an example, the peak location of Participant YO was 1 s, meaning that her expiration was 1 s behind the onset of the right-hand melody. This corresponds to the result found in the top panel of Figure 7B.

The peak value of cross correlation took large values for several participants though it was only marginal for the remaining participants. The magnitude of correlation reached a significant level for eight participants (*p* < 0.05). Therefore, at least for several participants, the breathing patterns were correlated with the musical structure of a contrapuntal music piece. On the other hand, the peak locations were distributed mainly around the range of 0–1 s. It may suggest that the expirations were likely to occur just after the onset of the right-hand subject.

#### Musical phrase

As another attempt to examine the relationship between musical organization and temporal breathing pattern, we also analyzed the PCO_2_ pattern for Excerpt E (Mozart, K.331).

This piece can be segmented into four “phrases” with a structure of A (4 measures), A′ (4 measures), B (4 measures), and A″ (6 measures): A is the main motif, A′ and A″ are the repeat/reproduction of A, and B is the development of A. If this phrase structure affects pianists' breathing, it can be speculated that the characteristics of respiration may be different between three A phrases and B phrase. In order to test this speculation, we compared the mean and variance of the expiration intervals between these four parts. In addition, we calculated the correlation coefficients between every pair of *avePCO*_2_(*t*)of these parts. Note that we did not perform this analysis for the last part (A″) because this part is an extended version of A and has two additional measures as a coda.

Resultantly, we failed to find any difference in mean and variance of the expiration intervals between these parts. In addition, there was no difference in the correlation coefficient between any pair of three parts. These results disconfirmed the speculation: breathing was not modulated by this phrase structure.

### Difference between participants

**Figure 8:**
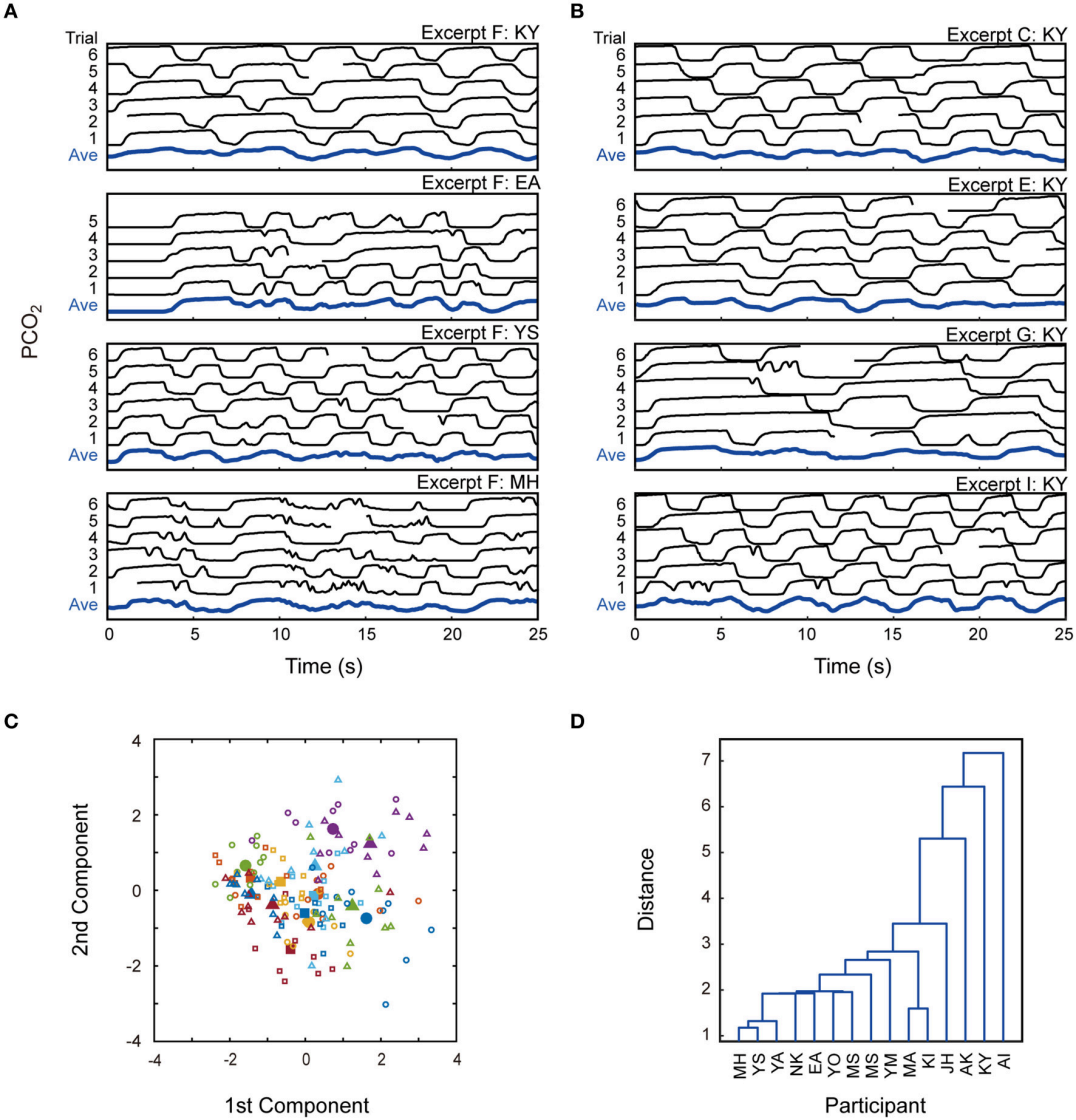
**Difference in breathing patterns between participants. (A)** Breath patterns of the first 25 s of Excerpt F for 4 participants are shown (result for another participant has been shown in Figure 3). Breathing pattern looks different between different pianists. **(B)** Breath patterns of one participant for Excerpt C, E, G and I are shown. These patterns generally look similar, suggesting that they shared features with each other. **(C)** Feature vectors of breathing patterns are plotted for each combination of the excerpts and participants (open symbols). The color of each symbol represents a participant. Filled symbols mean the inter-excerpt average feature vectors for individual participants. The same-colored symbols are distributed in a grouped manner, supporting that breathing patterns of individual pianists resembles between different music pieces. **(D)** Similarity of the respiratory pattern between the participants is shown by the dendrogram generated from the 5D feature vectors.

In the previous sections, we have repeatedly mentioned that the breathing patterns differed between individual participants. In order to illustrate this, we depict the PCO_2_ patterns of the first 25 s of Excerpt F for four participants in Figure 8A (data from another participant have been already shown in Figure 3).

Breathing patterns were considerably different between these participants: each participant exhibited characteristic features. To be more concrete, Participant KY breathed slowly and temporally-regularly. Another feature of his breathing is asymmetry between expiration and inspiration: the length of expiration phase was generally longer than that of inspiration phase. On the other hand, Participant EA apparently stopped breathing for a few seconds at the music onset, and Participant MH often showed jaggy breathing patterns. Such pianist-dependent tendency appeared commonly over different musical works. Figure 8B shows the PCO_2_ profiles of Participant KY for Excerpts C, E, G, and I. Although the absolute breath intervals varied depending on the musical works, the asymmetry between expiration and inspiration was consistently observed.

In order to quantify the uniqueness of breathing pattern of individual pianists, we calculated five parametric features for each combination of excerpts and pianists: (1) mean expiration interval, (2) CV of expiration interval, (3) SDAP, (4) duty ratio of expiration phase, and (5) mean of the first expiration time. Because absolute values of these parameters depended on musical works, we calculated their z-scores by normalizing them using their inter-participant means and standard deviations, separately for different excerpts. Then, we mapped 5D feature vectors onto a 2D plane using a principal component analysis (PCA). In Figure 8C, each open symbol indicates a combination of an excerpt and a participant, whose color represents a corresponding pianist. A filled symbol indicates the inter-excerpt average of the open symbols with the same color. The same-colored symbols are distributed close to each other, supporting the view that each pianist has his/her own breathing features and shared them between different musical works.

We performed a one-way MANOVA to test the effect of pianists on the feature vector. The effect was highly significant for all of four test statistics (Pillai, Wilks, Lawley-Hotelling, and Roy) of MANOVA (ps < 0.001). This supports that the respiratory patterns were different between the pianists. We depicted the dendrogram generated from the feature vectors (Figure 8D). A few participants are close to each other, but no clear cluster-structure can be found, implying that their respiratory patterns were generally different from one another.

### Answer to questionnaire

We obtained answers to the questionnaire from 14 participants.

#### Awareness of breathing in performance

Eleven participants answered that they were occasionally aware of their breathing in the performance. Among them, seven participants sometimes paid attention to their breathing in relation to musical expression. However, five participants cared about breathing with respect to performance anxiety and stress (i.e., rather than musical expression): They sometimes realized that they could not breathe normally (i.e., breathing stopped or shallowed) when they got nervous. One participant answered that she tried to make deep breaths when she felt nervous. On the other hand, two participants were afraid that noise of breathing might disturb the music sound.

#### Conscious control of breath timing

Seven participants answered that they sometimes adjusted their breathing consciously. Six among them changed their breathing according to the musical concepts and phrasing. For example, one participant described that she tried to breathe deeply and slowly for the slow, sublime pieces but quickly and briskly for fast, furious pieces. Another pianist answered that she sometimes adjusted her breath at the music onsets and phrase breaks. In addition, another participant tried to inhibit unnecessary breath in the middle of a long phrase. On the other hand, three among the seven pianists did not actively control the individual breath timings even if they paid attention to breathing.

#### Instruction on breathing in performance

All participants had experience of receiving instructions on breathing in performance. Twelve received advice on breathing in relation to musical expression: for example, to inhale at the onset of phrase, and to adjust breathing according to the musical concepts. The remaining participants noted that they were instructed not to stop breathing during performance.

#### Breathing in ensemble performance

All participants cared about the breath timing of other performers in ensemble performance. Twelve answered that they tried to adjust their own breathing to the others. In addition, one participant described that she could grasp how the other performers felt the music by attending to the depth and speed of their breathing.

#### Summary and supplementary points

The results of the above indicate that most participants paid attention to (and actively controlled) their breaths in specific situations, for example, when they felt anxiety, when they played specific parts and when they tried to get the right timing in ensemble performance. However, we should note that they were little aware of their breathing in usual performance except for such situations. Indeed, a few participants thought that it could be harmful to musical performance to be much aware of breathing. Especially, one participant gave a thought-provoking comment that the breathing was naturally determined as the result of performance, and not to be consciously controlled.

By contrast, three participants gave comments on the relationship between “musical gesture” and breathing. They thought that they could deliver their musical expression more effectively by signaling their breathing to the audience.

## Discussion

### Summary of results and their implications

The aim of the present study is to reveal the relationship between human respiratory function and musical performance, by analyzing the temporal breathing pattern in piano performance. Fifteen skilled pianists performed 10 music pieces while CO_2_ partial pressure (PCO_2_) was monitored around their mouth and nose. We analyzed several parametric and non-parametric features of temporal breathing patterns to extract the characteristic nature of respiratory timing in piano playing. We obtained the following novel findings.

First, mean breath interval was significantly modulated by the musical works. Generally, breathing was accelerated with faster tempi. We also found that breathing in piano performance was often slowed compared with the control (non-playing) condition. These results confirm that the respiration of the pianists is modulated by performance.

Second, breath intervals in piano playing were varied within a short period. Variation of the intervals depended on the musical works and the participants. Fluctuation of breath intervals was reduced for the pieces for finger exercise and those with faster tempi. This implies that the breathing was comparatively regular for the musically monotone pieces and for fast, rhythmic pieces. We also found a positive correlation between the inter-excerpt variation and within-trial fluctuation of breath intervals: the pianists displaying greater fluctuations within a music piece showed deeper modulation by the music works.

Third, we found that the inter-trial consistency of breathing patterns differed between the music works. The consistency was lower for Hanon's finger exercise and Debussy's *Clair de lune* and higher for the scales and Chopin's “Military Polonaise.” It was noteworthy that two finger exercise pieces (scale and Hanon's work) brought contrasting results. Indeed, one third of the participants showed quite regular breathing patterns correlated with ascending/descending sequence of the scale whilst such structured patterns were hardly found for the Hanon piece. This implies that even if a music work in the score looks musically colorless, the breathing pattern can be correlated to some organization/structure hidden in the piece.

Fourth, we analyzed the relationship between breath timing and musical events/organization. We found general tendencies that pianists exhaled just after the music onsets, inhaled at the rest notes, and inhibited inhale during the slur parts. As for a two-part contrapuntal piece (Bach's Invention), the breathing patterns of several participants correlated with the polyphonic structure. On the other hand, we failed to detect clear relationship between the breathing pattern and music structure for a Mozart's Sonata. Anyhow, we found that breathing in piano performance was surely modulated by the musical events and organization.

Finally, the present study revealed that the characteristics of respiratory patterns differed markedly among the pianists: every pianist showed their own specific features shared by different musical works though other parameters were different among them. We confirmed this by extracting parametric features from the temporal breathing pattern and mapping the feature vectors for every combination of excerpt and pianists. Feature vectors from the same pianist were located close to each other, meaning that the characteristics of breathing pattern were preserved for different music works.

The relationship between musical performance and respiration has been often mentioned and potentially accepted in the musician's community. Nevertheless, to our knowledge, there are only a few scientific reports describing the temporal respiratory patterns of pianists playing real musical works and analyzing them by scientific methods. The present study is the first systematic study that measures the respiratory patterns of many pianists playing a variety of musical works and analyzes their parametric and non-parametric characteristics. We are firmly convinced that the present work will promote scientific research on the relationship between physical and physiological aspects and musical performance.

In the following subsections, we would like to discuss more about the present findings in relation to the literature.

### Effect of musical parameter and organization on breathing

#### Tempo

King (2006) reported that breath rates in piano playing were comparatively higher than the base rate, but musical tempo did not necessarily influence breathing rate. By contrast, the present study showed that the breath frequency in piano playing can be either lower or higher than the control condition, and that the breathing was accelerated with faster tempi.

The difference between these studies is presumably due to the difference in the sample size. King (2006) dealt with only three pianists and three musical pieces, and found the different effects of musical works (i.e., tempi) among three pianists, which led to her conclusion. As we repeatedly noted, characteristics of breathing differed greatly between the pianists, and the effect of musical works was also significantly different between the participants (See Figure 2C). Therefore, it is natural that King (2006) reached the above conclusion. Contrastingly, the present study detected a positive correlation between the musical tempo and breathing frequency by analyzing the data from 15 pianists and 10 musical works.

As for the difference from the control (i.e., resting) condition, Ebert et al. (2002) also reported that mean breathing frequency in piano performance was higher than in resting. This seems contradictory to the present result that the breathing frequency in piano playing could be lower than in resting. However, considering that the task of their experiment was a Hanon-like finger exercise, it is possible that they made such an observation because the mean breath interval for the Hanon exercise (Excerpt B) was somewhat shorter than for the control condition also in the present study.

Therefore, the present finding is not contradictory to the previous findings. Rather, by using a larger number of pianists and musical works, the present result gives inclusive evidence about the relationship between the musical tempo and breathing rate.

#### Inter-trial consistency

The present study revealed that the inter-trial consistency of breathing patterns differed between the music works. This is one of the novel findings of the present study; this result was obtained by asking the pianists to perform every musical piece six times. Fundamentally, respiratory timing in piano performance must be unique for each performance. However, some features specific to musical pieces and pianists became observable when pianists played the same piece repeatedly.

It is interesting that a simple scale and a Hanon's work showed contrasting tendencies in spite that they are both monotonous, finger exercise pieces. A possible reason for the different tendencies for these pieces is the temporal length of their unit structures: the temporal length of ascending/descending sequence of the scale was close to the breath intervals, and entrainment or synchronization of breathing was more likely to occur; ascending and descending movements of Hanon's exercise may be too long for such entrainment. This idea could be tested by asking the pianists to play the same scale with different speeds.

As for the breathing in scale playing, Nassrallah et al. (2013) failed to find coordination or relation occurred between breathing and finger movement. However, they also reported that one (out of eight) participant showed a correlation. Considering that one third of the participants showed clearly structured breathing pattern in the present study, it is possible that majority of the participants showed no clear correlation in their study.

Another interesting result is that Debussy's *Clair de lune* showed a distinctive feature: Inter-trial consistency was markedly low, compared to the other works. At the present, the reason for this result is unclear. Further study, by examining the breathing pattern for other musical works by this composer, is needed to solve this issue.

#### Music onset, rest, and slur

As for the effect of the music onset, King (2006) reported that all of the performances were initiated by a “preparatory breath” before the music onset, and 72% of these breaths were inhalations. The present result showed the first expirations after the music onset were concentrated within 1 s after the music onset. This in turn means that most pianists finished inspiration before the music onset, and thus, this result is just in line with her result. On the other hand, she noted that the type of respiration (expiration vs. inspiration) at the start of the music was not consistent between different trials. However, this is not contradictory to the present result because not all expirations occurred just after the music onset.

We found that pianists tended to inhale at the rest notes and inhibit inhale during the slur parts. This is another novel finding of the present study. Considering that musical phrasing is a possible factor determining the breathing timing, as King (2006) pointed out, our finding is reasonable because rests can be generally linked to the beginning or end of a phrase, and slurs are located within a phrase: pianists tend to inhale around the phrase breaks.

#### Musical structure

We showed that the breathing patterns of several participants correlated with the subject vs. counter-subject structure of Bach's Invention. This correlation analysis seems the first attempt to reveal the effect of music structure.

On the other hand, we were not successful in detecting clear relationship between the breathing pattern and music structure for a Mozart's Sonata. This is surely a negative result, but it never denies the possibility that such musical structure modulates the temporal breathing pattern. The present experiment is our first attempt to analyze the relationship between respiration and musical performance, and thus, we used a variety of music works as the target pieces. In order to further pursue the relationship between musical structure and breathing, we should choose appropriate musical pieces for this purpose based on detailed music analysis. This is an important topic for future research.

### Inter-pianist difference in breathing pattern

The present study revealed that the breathing patterns were considerably different between the pianists. This inter-pianist variability has been pointed out by other researchers (King, 2006; Nassrallah et al., 2013). If human respiratory pattern is modulated by the motor and mental task (e.g., Boiten, 1993), it is possible that breathing patterns would be somewhat similar among different pianists because they perform the same motor task of playing the same musical piece. What factor brought the inter-pianist difference?

Several views may explain the inter-pianist variability. One is that the breathing in the piano performance is affected by body movement of the individual pianists (e.g., postural sway and head tilt). This is plausible because our respiration cannot be independent of the trunk movement. Such body movement is either by the necessity for sound production (e.g., for keeping appropriate arm posture) or by the musical gesture as a part of musical expression (e.g., Clarke and Davidson, 1998). Considering that body movements and gestures in piano performance are markedly different between pianists, it is possible that inter-pianist variability of breathing pattern may be related to variability of body movements.

Pushing the idea that the body movement is a part of musical expression, we can speculate that the differences of breathing pattern fundamentally stem from the difference in musical expression/interpretation of individual pianists. At the present, this is just a speculation and there is no objective support for this view. To test this view, we have to examine pianists' expression from various viewpoints, in relation to their musical interpretation. In this sense, music analysis of individual pianists must be essentially important: Correlation between the results of music analysis and the breathing patterns would be helpful to develop this discussion.

Another view is that respiratory patterns of individual pianists have been formed through their past experience. King (2006) termed pianist-specific features of breathing pattern “ingrained respiratory rhythm.” Although she did not mention how such patterns are ingrained, various (i.e., both musical and non-musical) experience could affect the current breathing pattern. It seems quite difficult to examine this view, but it may give a clue if we track the change in respiratory patterns of individual pianists for a long time.

### Breath control in piano playing

So far, we have not mentioned the underlying mechanism: how various factors affect the breathing in piano performance. As mentioned in the Introduction, our respiratory system basically operates via the neural oscillator system in our brainstem, whose activity is modulated by the signals from the cortical brain (e.g., Smith et al., 2013). Therefore, it is quite plausible that the respiration rhythm is altered by the music performance, which may be mainly achieved by the neural processing in the cortex. However, both the functional meaning and causal mechanism of this modulation are completely unknown. Why and how is our respiration correlated with musical performance? At present, we have no answer to this question, but we think that there are at least two possible views.

One is the view of “piano playing as singing.” This means that breath timing for singing corresponds to that for piano playing due to the fact that people originally treat music as a “song” even when playing instruments. This view was frequently expressed in informal discussion with the musicians. A shortcoming of this idea is that breathing for singing is essentially different from that for piano playing because singers have to keep expiring to make sounds. In fact, breath patterns of most participants were not directly synchronized with the melody in the present experiments. In this sense, musicians' “feeling of breath” may differ from the physiological respiration: perhaps, some performers may mean “flow of energy” inside their body by the term “breath.” Therefore, at least in the sense of respiration in singing, this view cannot explain the relationship between breathing and piano performance. Rather, we should consider that some factors accompanied by singing performance are related to piano performance, and they indirectly determined the respiration in piano playing.

Another view is the coupling at the motor control level. Not just in music performance, people usually inhale before taking an action (i.e., “preparatory breath”) and exhale in doing the action. This must be related to the physical fact that our respiration is achieved by contraction and relaxation of the diaphragm, and that motor action might be more freely executed by means of relaxing the respiratory muscles. Likewise, it seems natural that pianists inspire before the onset of new phrases, and expire in parallel with starting the phrase. In other words, the breath timing should be coupled with temporal organization of the body movement for music performance.

Because the key strokes for piano playing are much faster than the respiratory rhythm, the trunk, and head movements (rather than finger movements) are more likely to be related to the breathing function. In the pilot study by King (2006), however, the correspondence between physical movements and respiratory behavior was observed only one (out of three) pianist. Therefore, further examination is required to evaluate this view.

Besides these views, there may be possible explanations to the modulation of respiration in musical performance. Whatever the source of the modulation, we should keep in mind that such modulation is operated in a subconscious manner. In the questionnaire, most participants answered that they were little aware of their breathing in usual performance, excerpt for specific situations. This coincides with King's report (2006) that all of her participants were never conscious of their breathing in performance. Moreover, a few participants thought that it could be harmful to musical performance to be much aware of breathing. Therefore, the coupling between respiration and musical performance is unlikely to stem from the performers' conscious control of breathing.

### Further research topics on breathing in piano performance

The present paper introduced some characteristic features of breathing in piano playing. This is just the beginning of this research project and there remain a number of research topics.

One facile but interesting question is the breath patterns of world-famous artists. Do their breath patterns have some specific features? Are there similarities or differences between their patterns and the present results? We have plenty of questions on this subject. We are also interested in the breathing of musicians in a real concert and recital, because several participants commented in the questionnaire that breathing may be different between practice and concert.

The relationship between two or more musicians in an ensemble performance is also intriguing. Musicians generally recognize that their breath is an important cue for synchronizing the performance of multiple players, as the participants answered in the questionnaire. This point has been discussed by some researchers (e.g., McCaleb, 2014), and is surely an important issue.

Finally, it is necessary to expand and deepen the problems dealt with in the present study. As pointed out above, it must be informative if we compare the results of music analysis of different pianists and their breathing patterns. In addition, a more detailed examination on the effect of temporal parameters would be informative. Presumably, the breath pattern in playing the scale may be drastically changed if pianists are asked to play much slower or much faster.

Further researches on breath pattern in instrument playing will give us more understanding on the mechanism of breathing in music performance, together with some hints as to its role in a superb performance.

## Author contributions

YS planned this research and received grants from JSPS and from Yamaha for this topic. He designed and ran the experiment, analyzed the experimental data and drafted most part of the manuscript. EA built the experimental environment and gave essential ideas on the experimental procedure. She gathered the participants from the pianists' community and ran the experiment. She gave the interpretation of the experimental results especially from the viewpoint of pianists, and revised the manuscript.

## Funding

This work was supported by a Grant-in-Aid for Scientific Research (B) (#26280101) of Japan Society for the Promotion of Science (JSPS) and a research grant from Yamaha Music Foundation.

### Conflict of interest statement

The authors declare that the research was conducted in the absence of any commercial or financial relationships that could be construed as a potential conflict of interest.
